# (–)-Epigallocatechin-3-gallate induces apoptosis and differentiation in leukaemia by targeting reactive oxygen species and PIN1

**DOI:** 10.1038/s41598-021-88478-z

**Published:** 2021-04-27

**Authors:** Fernanda Isabel Della Via, Rodrigo Naoto Shiraishi, Irene Santos, Karla Priscila Ferro, Myriam Janeth Salazar-Terreros, Gilberto Carlos Franchi Junior, Eduardo Magalhães Rego, Sara Teresinha Olalla Saad, Cristiane Okuda Torello

**Affiliations:** 1 Haematology and Transfusion Medicine Centre – Hemocentro, University of Campinas, Campinas, 13083-878 Brazil; 2 Onco-Haematological Child Centre, Faculty of Medical Sciences, University of Campinas, Campinas, 13083-970 Brazil; 3 Haematology and Clinical Oncology Division, Department of Internal Medicine, University of São Paulo, Ribeirão Preto, 14048-900 Brazil

**Keywords:** Cancer models, Acute myeloid leukaemia

## Abstract

(–)-Epigallocatechin-3-gallate (EGCG), the major active polyphenol extracted from green tea, has been shown to induce apoptosis and inhibit cell proliferation, cell invasion, angiogenesis and metastasis. Herein, we evaluated the in vivo effects of EGCG in acute myeloid leukaemia (AML) using an acute promyelocytic leukaemia (APL) experimental model (PML/RARα). Haematological analysis revealed that EGCG treatment reversed leucocytosis, anaemia and thrombocytopenia, and prolonged survival of PML/RARα mice. Notably, EGCG reduced leukaemia immature cells and promyelocytes in the bone marrow while increasing mature myeloid cells, possibly due to apoptosis increase and cell differentiation. The reduction of promyelocytes and neutrophils/monocytes increase detected in the peripheral blood, in addition to the increased percentage of bone marrow cells with aggregated promyelocytic leukaemia (PML) bodies staining and decreased expression of PML-RAR oncoprotein corroborates our results. In addition, EGCG increased expression of neutrophil differentiation markers such as CD11b, CD14, CD15 and CD66 in NB4 cells; and the combination of all-trans retinoic acid (ATRA) plus EGCG yield higher increase the expression of CD15 marker. These findings could be explained by a decrease of peptidyl-prolyl isomerase NIMA-interacting 1 (PIN1) expression and reactive oxygen species (ROS) increase. EGCG also decreased expression of substrate oncoproteins for PIN1 (including cyclin D1, NF-κB p65, c-MYC, and AKT) and 67 kDa laminin receptor (67LR) in the bone marrow cells. Moreover, EGCG showed inhibition of ROS production in NB4 cells in the presence of N-acetyl-L-cysteine (NAC), as well as a partial blockage of neutrophil differentiation and apoptosis, indicating that EGCG-activities involve/or are in response of oxidative stress. Furthermore, apoptosis of spleen cells was supported by increasing expression of BAD and BAX, parallel to BCL-2 and c-MYC decrease. The reduction of spleen weights of PML/RARα mice, as well as apoptosis induced by EGCG in NB4 cells in a dose-dependent manner confirms this assumption. Our results support further evaluation of EGCG in clinical trials for AML, since EGCG could represent a promising option for AML patient ineligible for current mainstay treatments.

## Introduction

(–)-Epigallocatechin-3-gallate (EGCG) is a gallate ester obtained by the condensation of gallic acid with the (3R)-hydroxy group of the catechin (–)-epigallocatechin. EGCG is considered the most biologically active and abundant catechin found in green tea, accounting for at least 50% of the total amount of catechins^1–3^. Green tea (*Camellia sinensis*) represents 20% of the total tea produced and consumed worldwide^4,5^; production involves steaming or pan-frying the freshly harvested leaves, in order to inactive enzymes such as polyphenols oxidase thus preserving the active chemical polyphenolic characteristics of catechins^3,5,6^. Epidemiologic studies have linked green tea consumption to a decreased risk of cancer^7–9^. Inhibition of tumour formation and growth due to the presence of catechins has also been described in animal models^5^. EGCG is known to have multiple transduction pathways and enzyme activities that could induce apoptosis, and suppress cell proliferation, invasion, angiogenesis and metastasis in cancers.

Haematological cancers remain a global health problem. Acute myeloid leukaemia (AML) is a clinically heterogeneous haematological malignancy, characterized by abnormal proliferation of immature myeloid progenitors resulting in bone marrow failure^10^. AML is commonly regarded as the result of genetic changes in haematopoietic stem cells leading to an irreversible dysregulation of critical gene functions such as differentiation, proliferation and apoptosis^11^. Despite the fact that advances in the treatment of AML have led to better survival of young patients, the treatment for elderly patients, who represent the majority of the new cases, remains a challenge since these patients present worse survival and are ineligible for aggressive therapies or for bone marrow transplantation^12^. Recently, hypomethylating agents such as azacytidine have been the treatment option for these patients inducing haematological improvement and prolonging survival; however, positive haematological responses occur in only 30% of elderly patients^13^. In this context, the search for new treatment options for these AML patients remains a challenge.

Green tea and EGCG have been reported to present anti-proliferative and pro-apoptotic effects in myeloid leukaemia cell lines^1,14–16^ as well as *in vivo* in AML xenografts^14,15^. Several molecular targets for EGCG have been reported, but the mechanisms of its anticancer activities are not yet clearly understood. EGCG can induce the production of reactive oxygen species (ROS) in cancer cells and induce apoptosis, and paradoxically may act as an antioxidant, decreasing ROS and inhibiting cancer development^17^. EGCG is also known to bind and modulate the activities of enzymes, receptors, and signalling molecules that affect cell growth and proliferation^18^. EGCG can inhibit peptidyl-prolyl isomerase NIMA-interacting 1 (PIN1) activity, an enzyme that binds to and catalyses the conversion of proline-directed serine/threonine phosphorylation, could disrupt the balance of oncogenes and tumour suppressors promoting oncogenesis^19–21^, which point out the therapeutic potential of PIN1 inhibitors in cancer therapy. EGCG is also described to bind 67 kDa laminin receptor (67LR) inducing cancer cell apoptosis^22^.

We previously reported that green tea (whole extract) decreased leucocytosis and promoted a reduction of immature cells in the bone marrow and spleen of PML/RARα mice by inducing apoptosis^23^. As the *in vivo* effects of isolated EGCG in AML have not yet been evaluated, the aim of this study was to evaluate the molecular mechanisms involved in anti-leukaemia activity of EGCG using the acute promyelocytic leukaemia (APL) model (PML/RARα). This is a well-established transgenic model of APL^23–25^. APL is a subtype of AML characterized by a specific chromosomal translocation, t(15;17) that encodes a fusion of the proteins of promyelocytic leukaemia (PML) and retinoic acid receptor-α (RARα)^26^. The oncoprotein PML/RARα drives to deregulation of transcription, differentiation arrest, and enhanced self-renewal of leukaemia-initiating blast cells^27,28^.

## Results

### EGCG ameliorates the haematological parameters and prolonged survival of PML/RARα mice

**Figure 1:**
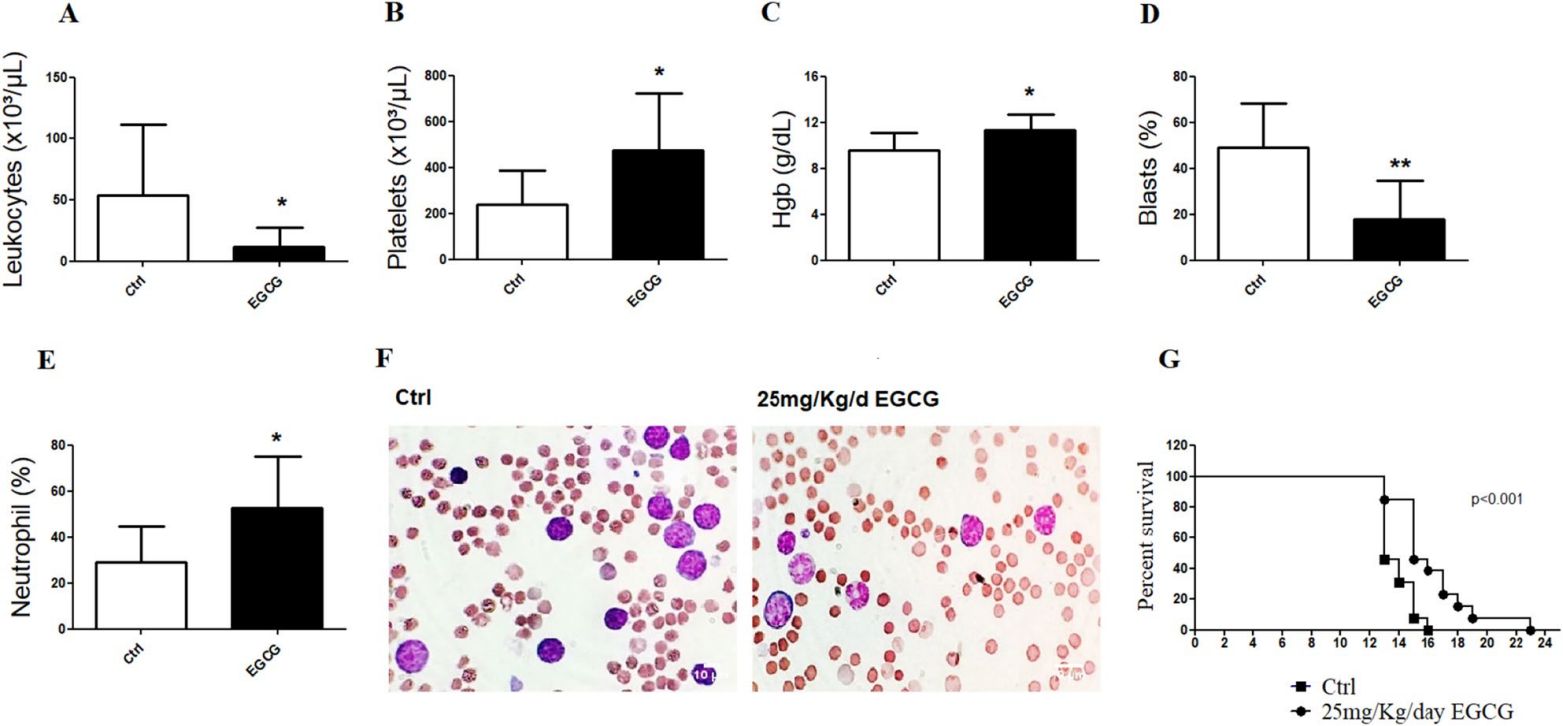
Haematological data of PML/RARα mice submitted to EGCG treatment. At the end of treatment (16th day) with EGCG (25 mg/kg/day i.p.) or vehicle (Ctrl), PML/RARα mice were bled from the retro-orbital plexus. EGCG induced a reduction of leukocytes (**A**), and an increase of platelets (**B**) and haemoglobin levels (**C**) using the CELL-DYN Emerald Haematology System counter. A reduction of blasts percentage (**D**) and an increase of neutrophils percentage (**E**) were also observed in the differential counts obtained from peripheral blood Leishman–Wright–Giemsa-stained smears. Representative images of peripheral blood Leishman–Wright–Giemsa-stained smears (100 ×) of PML/RARα mice treated with vehicle (Ctrl) or EGCG (**F**). EGCG treatment also increased the survival of PML/RAR mice (**G**). Statistical significance (Student t test) is indicated, as follows: *P < 0.05; ** P < 0.01. For survival curve statistical significance (Log-Rank; Mantel-cox test) is indicate, as follow: *P* < 0.001.

EGCG treatment of PML/RARα mice decreased leukocytes number whereas increased platelets number and haemoglobin levels (Fig. 1A–C). In addition, differential counts obtained from peripheral blood Leishman-Wright-Giemsa-stained smears showed a reduction in the percentage of blasts parallel to an increase of neutrophils (Fig. 1D–F). Moreover, PML/RARα mice treated with EGCG showed a significant (*P* < 0.001) longer survival than vehicle-treated mice (Fig. 1G).

### EGCG reduces leukaemia immature cells and promyelocytes in the bone marrow, spleen and peripheral blood

**Figure 2:**
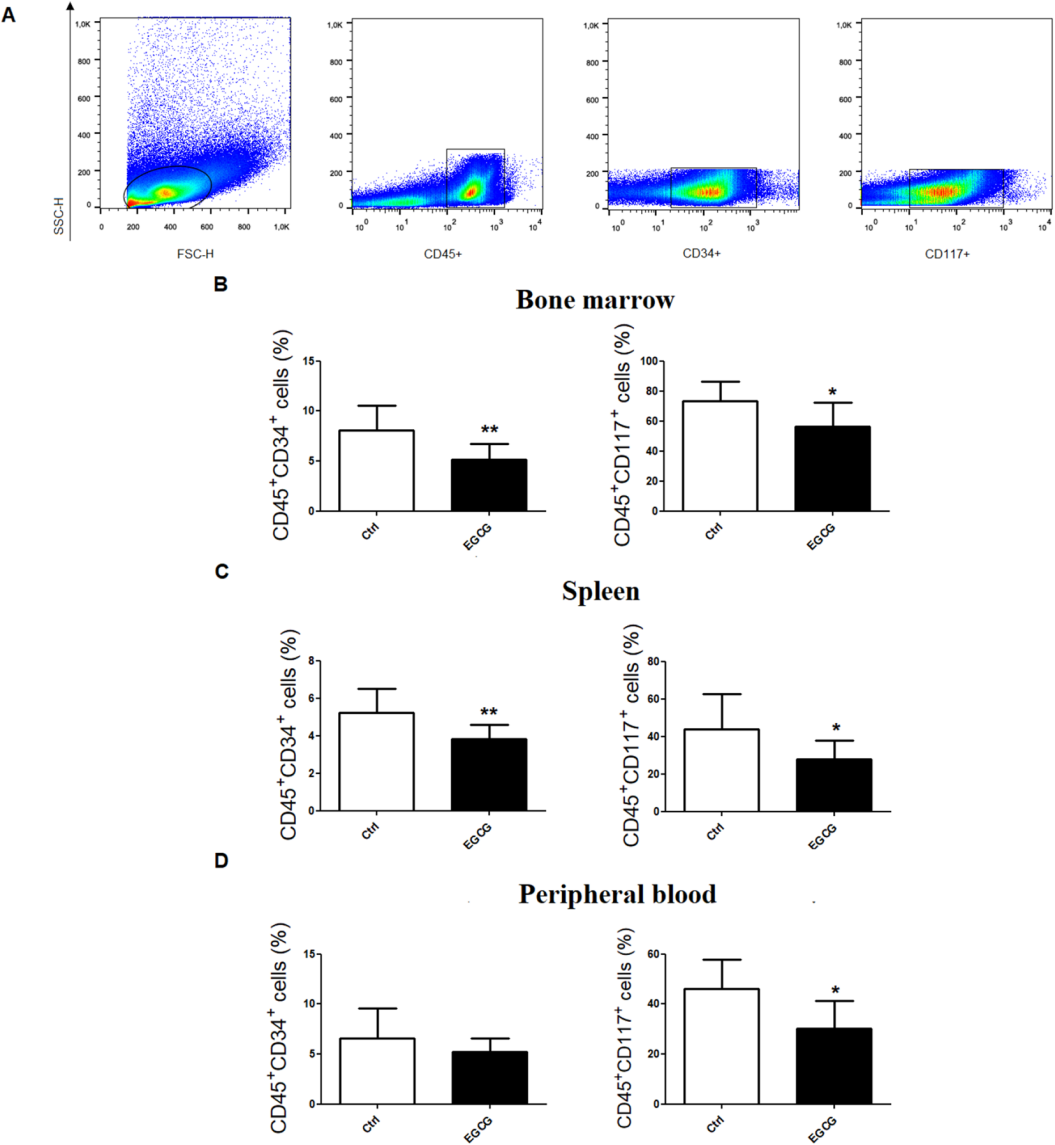
EGCG treatment reduced leukaemia immature cells in bone marrow, spleen and peripheral blood. At the end of treatment (16th day) with EGCG (25 mg/kg/day i.p.) or vehicle (Ctrl), total bone marrow and spleen cells of PML/RARα mice were incubated with CD45 to exclude erythroid lineage, and then CD34 and CD117 antibodies were used to detect hematopoietic stem cells and promyelocytes, respectively (**A**); EGCG induced a reduction of CD45^+^CD34^+^ and CD45^+^CD117^+^ percentage in bone marrow (**B**) and spleen (**C**), as well as decreased CD45^+^CD117^+^ percentage in the peripheral blood, with no changes in CD45^+^CD34^+^ cells (**D**). Statistical significance (Student *t* test) is indicated as follows: * P < 0.05; ** P < 0.01.

Total cells from the bone marrow, peripheral blood and spleen of PML/RARα mice were incubated with CD45, CD34 and CD117 markers. The percentage of CD45 positive cells were selected to exclude erythroid lineage, and subsets were then generated to evaluate the expression of CD34 (hematopoietic stem cells) and CD117 (promyelocytes) markers (Fig. 2A). The CD34 marker represents leukaemia stem cells, typically observed in AML^29^; the CD117 marker is the c-kit proto-oncogene encoding the receptor tyrosine kinase involved in the proliferation of leukaemia cells found in the stage one of myeloid differentiation^30^. EGCG treatment reduced the percentage of CD45^+^CD34^+^ cells in the bone marrow (Fig. 2B) and spleen (Fig. 2C) of PML/RARα mice, and also reduced the percentage of CD45^+^CD117^+^ cells in the bone marrow (Fig. 2B) and spleen (Fig. 2C). In the peripheral blood, we observed a reduction of CD45^+^CD117^+^, with no changes in CD45^+^CD34^+^ cells (Fig. 2D).

### EGCG induces cellular differentiation

**Figure 3:**
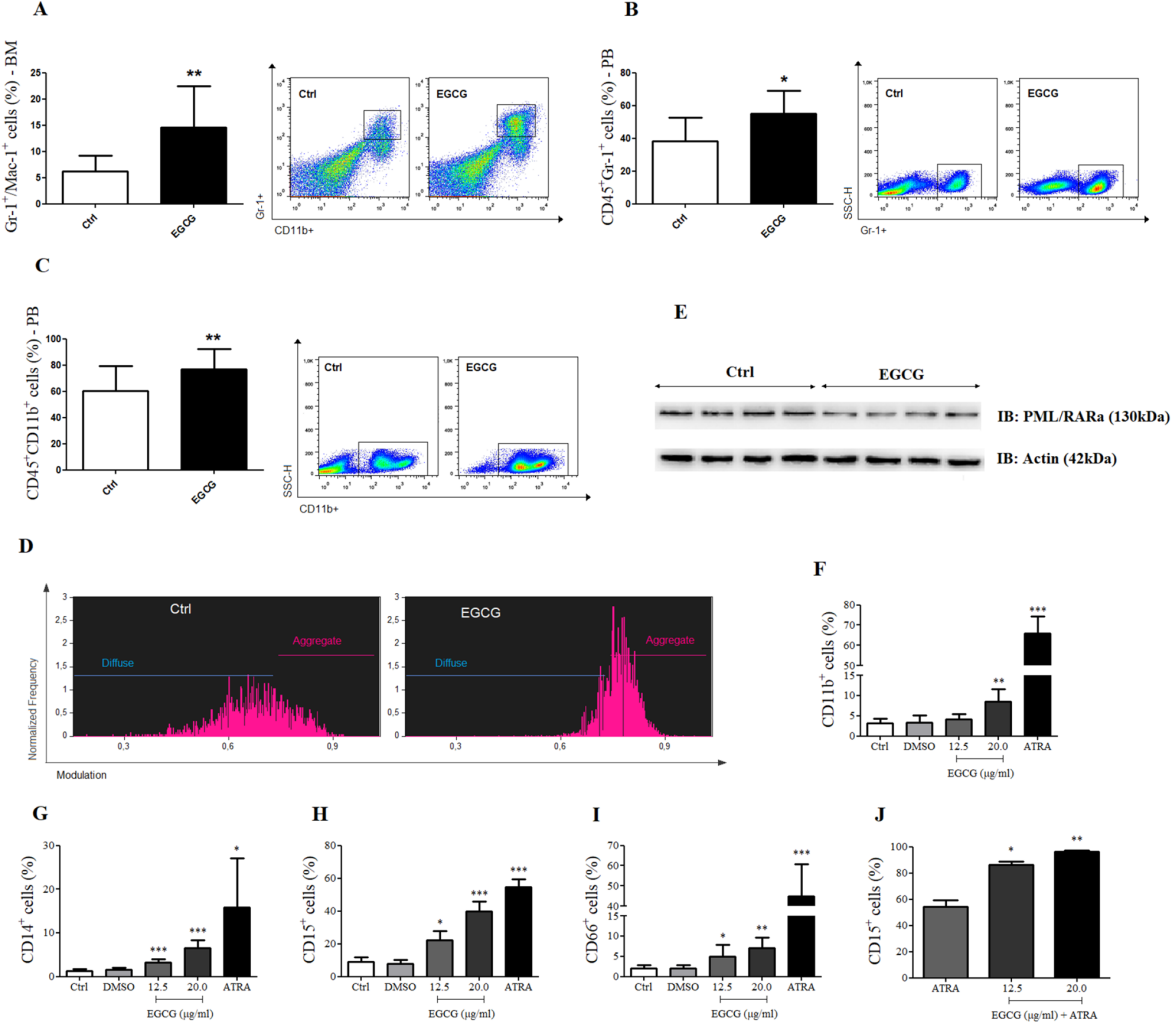
EGCG induced cell differentiation in bone marrow cells. At the end of treatment (16th day) with EGCG (25 mg/kg/day i.p.) or vehicle (Ctrl), bone marrow (BM) and peripheral blood (PB) cells of PML/RARα mice were submitted to analysis. EGCG increased myeloid cells (CD11b^+^Gr-1^+^) percentage in bone marrow (**A**) and increased the percentage of granulocytes (CD45^+^Gr-1^+^) (**B**) and monocytes (CD45^+^CD11b^+^) (**C**) in peripheral blood, analysed by flow cytometry. EGCG decreased diffuse staining of promyelocytic leukaemia (PML) bodies in bone marrow cells, parallel to an increase in aggregated stain detected by image flow cytometry (**D**). EGCG decreased PML/RARα expression in BM cells detected by western blotting (**E**). ECGC increased the percentage of neutrophil differentiation markers: CD11b, CD14, CD15 and CD66 in NB4 cells (**F**–**I**); and the combination of ATRA and EGCG yield higher increase the expression of CD15 marker (**J**); three independents experiments. Gels were run under the same experimental conditions and the images of western blots displayed in cropped format. Full-length blots/gels are presented in Supplementary Fig. S1. Statistical significance (Student *t* test) is indicated, as follows: * P < 0.05; ** P < 0.01; *** P < 0.0001.

EGCG treatment also increased the percentage of myeloid cells (CD11b^+^Gr-1^+^) in the bone marrow of PML/RARα mice (Fig. 3A). Corroborating these findings, we further observed an increase of circulating granulocytes (CD45^+^Gr-1^+^ cells) (Fig. 3B) and monocytes (CD45^+^CD11b^+^ cells) (Fig. 3C). To support these results, we studied EGCG effects on PML bodies of total bone marrow cells of PML/RARα mice by imaging flow cytometry. EGCG treatment decreased diffuse staining of PML bodies in the bone marrow cells of PML/RARα mice while increasing aggregated staining (Fig. 3D). In addition, decreased expression of PML/RAR oncoprotein was detected in the bone marrow cell lysates of PML/RARα mice by western blotting (Fig. 3E). Furthermore, *in vitro* assays performed in NB4 cells corroborate our *in vivo* results showing that EGCG treatment (12.5–20.0 µl), similarly to all-trans retinoic acid (ATRA; the drug approved for APL therapy), increased the expression of neutrophil differentiation markers such as CD11b, CD14, CD15 and CD66 in NB4 cells in a dose-dependent manner after 96 hours incubation (Fig. 3F). It is important to mention that EGCG is less potent than ATRA, however the combination ATRA plus EGCG yields a higher increase in the expression of CD15 marker (Fig. 3G).

### EGCG induced differentiation by decreasing PIN1 expression and ROS increase

**Figure 4:**
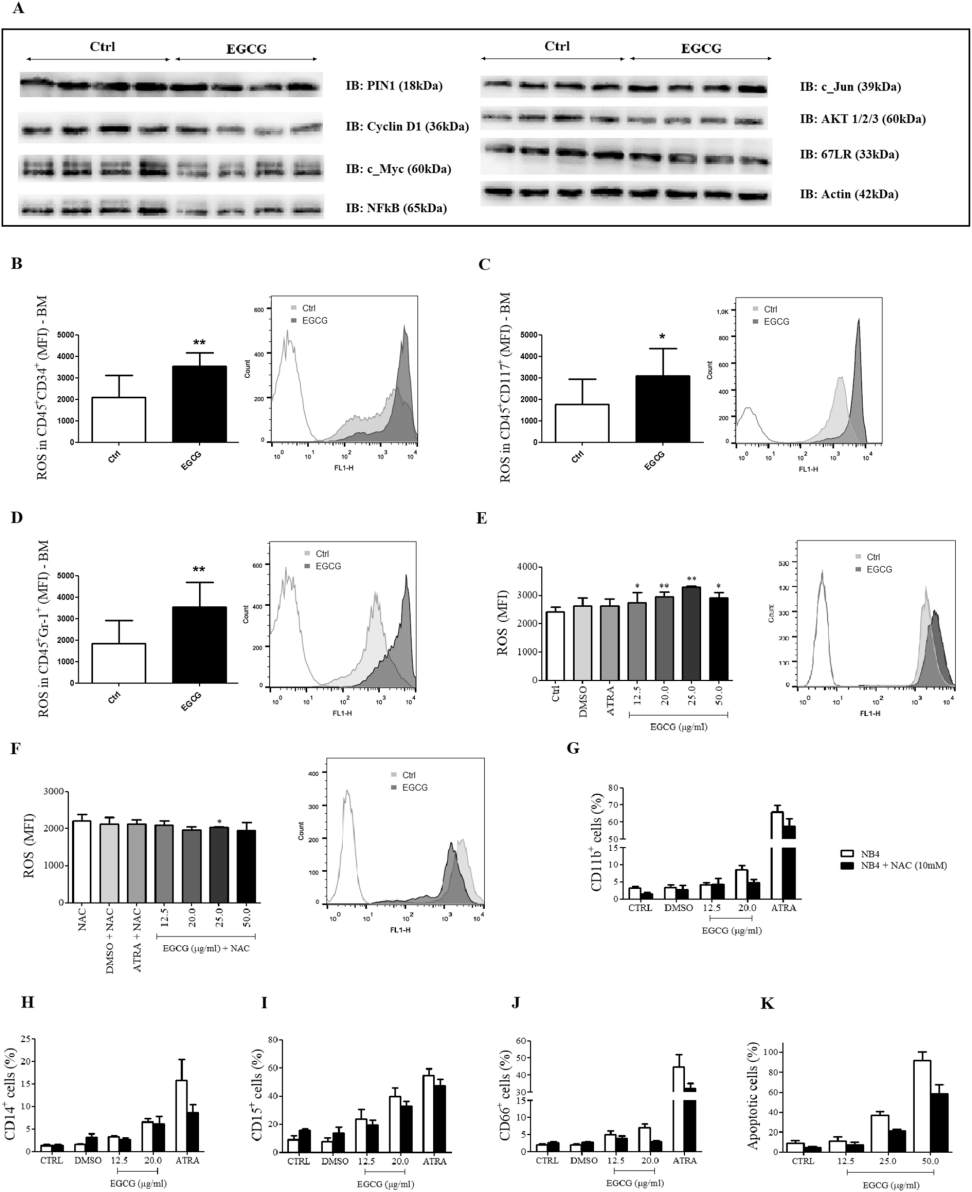
EGCG decreased PIN1, Cyclin D1, c-Myc, NFκB, 67LR, and AKT expression in bone marrow (BM) cells detected by western blotting and no difference was found in c-Jun expression (**A**). EGCG induces an increase of intracellular reactive oxygen species (ROS) in bone marrow cells. At the end of treatment (16th day) with EGCG (25 mg/kg/day i.p.) or vehicle (Ctrl), bone marrow cells of PLM/RARα mice were incubated with CD45, CD34, CD117, CD11b and Gr-1 antibodies, and 2′,7′-dichlorofluorescein diacetate (DCFDA) to determine the mean florescence intensity (MFI) of ROS. EGCG induced an increase in MFI of ROS in immature cells CD45^+^CD34^+^ (**B**) and CD45^+^CD117^+^ (**C**), and in granulocytes CD45^+^Gr-1^+^ (**D**). EGCG induced an increase in MFI of ROS in NB4 cells (**E**), and the addition of NAC (10 mM) attenuated this effect (**F**); three independents experiments. ECGC led to a partial blockage of neutrophil differentiation (Fig. 4G–J) and apoptosis (Fig. 4K) in NB4 cells in the presence of NAC; three independents experiments. Gels were run under the same experimental conditions and the images of western blots displayed in cropped format. Full-length blots/gels are presented in Supplementary Figs. S2–S3. Statistical significance (Student *t* test) is indicated as follows: * P < 0.05; ** P < 0.01.

To understand the molecular mechanisms involved in anti-leukemic activity of EGCG, we investigated PIN1, the substrate oncoproteins for PIN1 (including cyclin D1, NF-κB p65, c-MYC, c-Jun and AKT), and 67LR. EGCG treatment induced a decrease in the expression of PIN1, cyclin D1, NF-κB p65, c-MYC, AKT and 67LR in the bone marrow cell lysates (Fig. 4A), with no effects in c-Jun expression. In addition, since oxidative stress is important for APL treatment and EGCG displays both pro-oxidant and anti-oxidant effects, we investigated the role of EGCG in ROS levels of the bone marrow cells. EGCG increased mean fluorescence intensity (MFI) of ROS in CD45^+^CD34^+^ cells (Fig. 4B), in CD45^+^CD117^+^ cells (Fig. 4C), and in CD45^+^Gr-1^+^ granulocytes (Fig. 4D). Supporting these findings, we observed an increase in ROS after 2 hours treatment of the NB4 cells with EGCG (12.5–50.0 μg/ml) (Fig. 4E). Furthermore, treatment of the NB4 cells with the antioxidant N-acetyl-L-cysteine (NAC), together with EGCG, induced inhibition of the intracellular ROS production (Fig. 4F), as well as led to a partial blockage of neutrophil differentiation (Fig. 4G-J) and apoptosis (Fig. 4K), compared to NB4 cells treated with EGCG alone, indicating that EGCG-activities involve/or are in response of oxidative stress.

### EGCG increases apoptotic cells in the spleen by modulating BAX, BAD, BCL-2 and c-MYC

**Figure 5:**
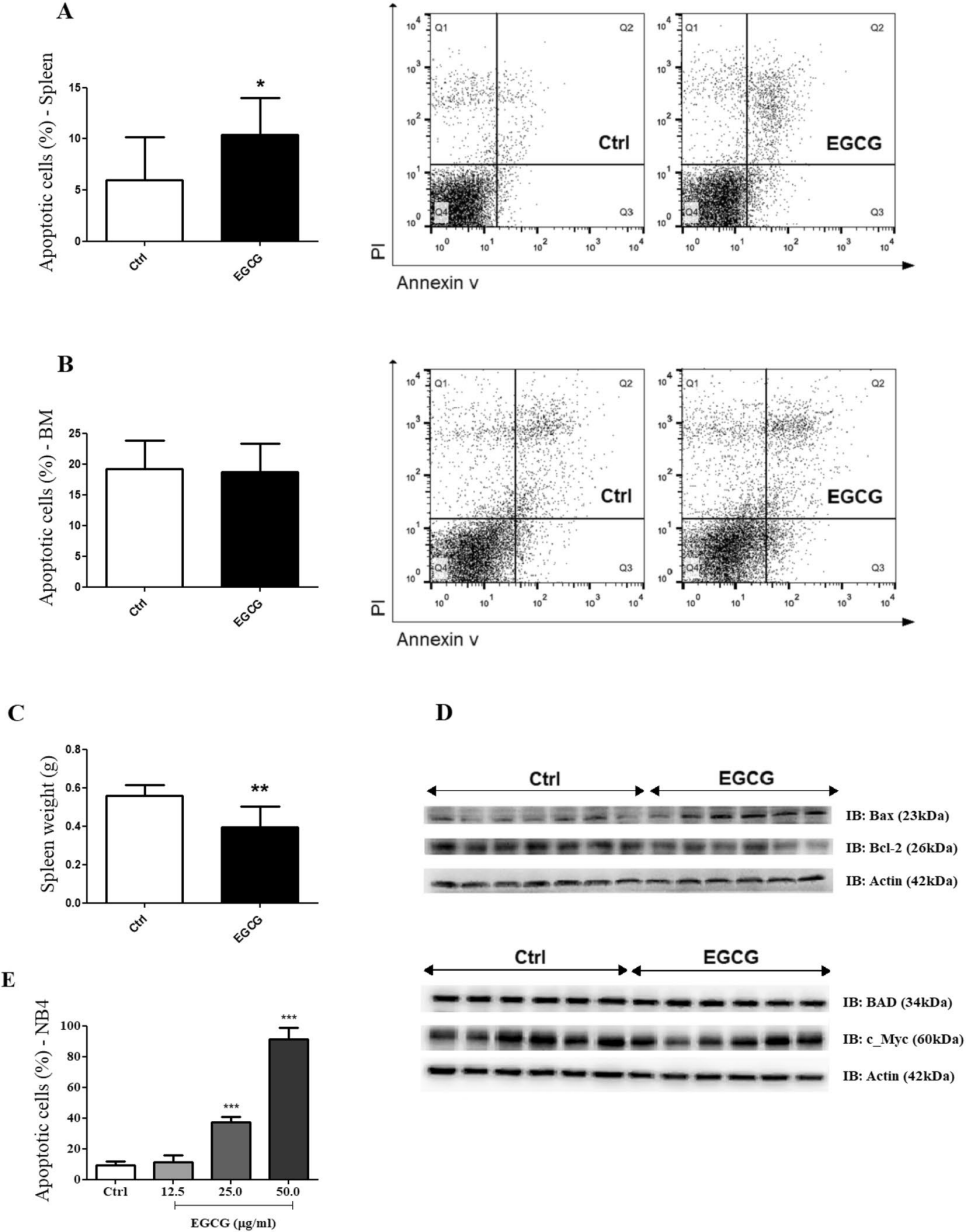
EGCG induces apoptosis of spleen cells by regulating BAX, BAD, BCL-2 and c-MYC expression. At the end of treatment (16th day) with EGCG (25 mg/kg/day i.p.) or vehicle (Ctrl), bone marrow (BM) and spleen cells of PLM/RARα mice were analysed by flow cytometry using Annexin-V/Propidium iodide and western blot. EGCG induced an increase of apoptosis in spleen cells (**A**), with no changes in bone marrow (**B**); representative dot plots of apoptosis analysis on the right. EGCG reduced spleen weights of PML/RARα mice (**C**) and increased expression of BAX and BAD whereas decreasing expression of BCL-2 and c-MYC (**D**). EGCG induced an increase in apoptosis in NB4 cells in a dose-dependent manner (**E**); three independents experiments. Gels were run under the same experimental conditions and the images of western blots displayed in cropped format. Full-length blots/gels are presented in Supplementary Figs. S4 and S5. Statistical significance (Student *t* test) is indicated as follows: * P < 0.05; ** P < 0.01; *** P < 0.0001.

As previously reported, green tea extract reduced leucocytosis and immature cells in the bone marrow and spleen of PML/RARα mice by inducing apoptosis^23^. Thus, we investigated cell death by apoptosis in the bone marrow and spleen cells to verify whether isolated EGCG displays this ability. EGCG treatment induced a significant increase of apoptotic cells in the spleen of PML/RARα mice (Fig. 5A) and no difference was found in the bone marrow cells (Fig. 5B). Consistent with these findings, EGCG treatment also significantly reduced spleen weights of PML/RARα mice (Fig. 5C). To support these results, we next evaluated the expression of BAX, BAD, BCL-2 and c-MYC proteins in the spleen cells lysate. EGCG treatment increased expression of BAX and BAD while decreasing expression of BCL-2 and c-MYC (Fig. 5D). Moreover, the *in vitro* results further demonstrated that EGCG (12.5–50 µg/ml) induced cell death in NB4 cells by increasing the percentage of apoptotic cells in a dose-dependent manner (Fig. 5E).

## Discussion

In the present study, we investigated the effect of EGCG on the well-established transgenic model of APL^23–25^. Treatment of PML-RARα mice with EGCG (25mg/kg/day) for five consecutive days prolonged survival of mice and reduced leucocytosis, anaemia, and thrombocytopenia. Earlier results of the same mice model treated with green tea extract showed a reduction of leucocytosis only, suggesting that EGCG alone could be more efficient in APL when compared to the whole extract from green tea^23^. In this sense, we evaluated EGCG effect on leukaemia immature cells of PML-RARα mice. Reductions of CD45^+^CD34^+^ and/or CD45^+^CD117^+^ cells were observed in the bone marrow and peripheral blood of PML/RARα mice treated with EGCG. Leukaemia cells of PML/RARα transgenic mice have been well established to display positive expression of CD34 and CD117 antigens^23,25^. The CD34 represents the leukaemia stem cells, typically observed in acute myeloid leukaemia^29^; the CD117 is the c-kit proto-oncogene encoding the receptor tyrosine kinase involved in the proliferation of leukaemia cells found in stage one of myeloid differentiation^30^. EGCG treatment also increased the number of mature cells (CD11b^+^/Gr-1^+^) in bone marrow, and the number of circulating neutrophils and monocytes, suggesting that EGCG induced cell differentiation. To support these findings, we demonstrated that EGCG treatment decreased the expression of the PML/RARα oncoprotein and decreased the diffuse stain of PML bodies in the bone marrow cells while increasing aggregated stain of PML bodies, indicating a higher degradation of PML/RARα oncoprotein by EGCG. According to Grimwade^31^, normal cells have an aggregated stain of PML bodies whereas leukaemia cells have a diffuse staining. Moreover, our *in vitro* results showed increased expression of neutrophil differentiation markers such as CD11b, CD14, CD15 and CD66 in NB4 cells after treatment with EGCG; and the combination of ATRA plus EGCG yield higher increase the expression of CD15 marker. These results are consistent with previous data reporting the ability of EGCG to induce leukaemia cell differentiation. Studies *in vitro* have demonstrated that EGCG upregulated the expression of differentiation markers (CD11B and CD15) and differentiation-inducing genes (CEBPE and CSF3R); the co-treatment of APL cells with ATRA plus EGCG enhanced neutrophil differentiation^1^, and EGCG further decreased the expression of PML/RARα in these cells^14,32,33^. Wei et al.^21^ demonstrated *in vivo* that a lower dose of EGCG (12.5 mg/kg) than the one used in our study (25 mg/kg) did not increase mature cells despite effectively reducing PML/RARα protein expression in the bone marrow of animals in pre-leukaemia phase.

A possible mechanism that could explain EGCG effect on differentiation is related to its action on PIN1. PIN1 is involved in controlling the activity and stability of proteins^34^. In cancer, PIN is overexpressed and/or over activated^21^, correlating with poor outcomes. PIN1 upregulates >50 oncogenes or proliferation-promoting factors while inhibits >20 tumour suppressors or proliferation-restraining factors^20^. In case of APL, PIN1 stabilize the PML/RARα oncoprotein and the genetic or chemical ablation of PIN1 leads to PML/RAR degradation, thus being effective in treating APL mice or patients^20,21^. There is evidence that EGCG is capable of binding to both functional domains of PIN1, WW and PPIase, leading to the suppression of tumour-promoting activity of PIN1^35^. PIN1 has the property of binding to, and catalysing the conversion of proline-directed serine/threonine phosphorylation^20^, common and central signalling mechanism in oncogenic pathways^21^. In this context, we observed a reduction of cyclin D1, NF-κB p65, c-Myc and AKT proteins in bone marrow cell lysates of PML/RARα mice after EGCG treatment, those all considered oncogenic proteins for PIN1. The literature proposes a correlation between PIN1 and cyclin D1, in which PIN1 increases cyclin D1 transcription directly or by Jun N terminal Kinase and/or cytokine-nuclear factor (NF)-κB pathways, resulting in increased proliferation of cancer cells^20,36^. AKT (also called protein kinase B) is another oncogenic protein stabilized by PIN1. PIN1 isomerization of AKT is critical for activation of the AKT signalling cascade that in turn activates the transcription of genes encoding cyclin D1. In cancer cells, high levels of PIN1 amplify the activation of the AKT cascade thus enhancing tumour progression^37^. Another interesting finding was the reduction of c-Myc expression by EGCG, a transcription factor co-overexpressed with PIN1 in human cancer cells^38^. PIN1 can positively regulate c-Myc transcriptional activity and promoter binding, and this sustained activation of c-Myc can inhibit terminal differentiation^39^. Moreover, we demonstrated herein the decreased expression of 67LR after EGCG treatment, which corroborates with literature data showing decreased expression of these receptor (commonly overexpressed in leukaemia cells) with the ATRA-induced neutrophil differentiation, in NB4 and HL60 cells^1,40^. The 67LR receptor is a non-integrin cell surface receptor for laminin with high affinity^41^ and the expression level of the protein correlates with the basement membrane invasion and metastasis of cancer cells^1,40^ as well as the drug resistance^42^. Interesting, the 67LR has been identified as surface receptor for EGCG activities^2,41,42^.

Another mechanism that could be related to EGCG action on differentiation is the pro-oxidant profile of EGCG. We found increased levels of ROS in immature cells and neutrophils of PML-RARα mice after EGCG treatment. This results are consistent with the literature data demonstrating that EGCG induces the generation of oxidative stress (ROS formation) *in vitro* using APL cells, B cells and H1299 cells, as well as *in vivo*, in xenograft mice^14,15,43^. In previous work, we demonstrated that 250 mg/kg/day of green tea extract also increased ROS in neutrophils while decreasing their levels in immature cells^23^. This difference could be attributed to some additional component found in green tea as the whole extract was used. In addition, EGCG has been well established to be auto-oxidized under cell culture conditions, leading to the formation of ROS molecules, such as superoxide radicals and hydrogen peroxide^44^. In this respect, various drugs, such as anthracyclines and arsenic trioxide, have been used for leukaemia therapy and their mechanism of action involve ROS generation^45^. In APL, the induction of ROS by arsenic trioxide is a critical regulator both for the biogenesis of PML nuclear bodies and PML/RARα degradation^27^. Our *in vitro* results showed inhibition of ROS production by treatment of the NB4 cells with NAC plus EGCG, as well as a partial blockage of neutrophil differentiation and apoptosis, indicating that EGCG-activities involve/or are in response of oxidative stress. A down-regulation of ROS by antioxidants, such as NAC, blocked the differentiation of APL cell line, while an over-expression of ROS increased cell differentiation has been previously described^46^. Moreover, in the murine APL model, ROS-inducers lead to PML/RARα degradation, regression of the disease and/or longer survival^47^; our prolonged survival of PML/RARα mice after EGCG treatment corroborates these assumption.

The reduction of immature cells found in the spleen cells could be explained by the increase of apoptotic cells and regulation of BCL-2 family proteins (EGCG reduced BCL-2 expression, whereas increased BAX and BAD expression), and c-Myc protein. These findings are in accordance with literature data showing that green tea catechins has the anti-leukaemia activity mainly due to the induction of apoptosis *in vitro*^1,14–16^ and *in vivo*, both in murine xenograft model^14,15,48,49^ and in APL model^23^. EGCG could affect apoptosis by modulating the level of expression of anti-apoptotic BCL-2 or pro-apoptotic BAX and BAD proteins^50^. In addition, BCL-2 is an apoptotic target suppressed by c-Myc ^51^, thus supporting apoptosis induced by EGCG. Moreover, these findings were corroborated by the reduction of spleen weights of PML/RARα mice after EGCG treatment, as well as the apoptosis induced by EGCG in NB4 cells in a dose-dependent manner.

Collectively, our results indicate that EGCG reduced leukaemia burden, induced apoptosis and differentiation thus resulting in a longer survival of PML/RARα mice. This was explained by EGCG ability to modulate oxidative stress activity inducing ROS production and bind to molecules leading to inhibition of enzymes activities (PIN1), modulation of signalling molecules (BCL-2, BAX, BAD, Cyclin D1, c-Myc, NF-κB p65, AKT) and modulation of receptors function (67LR), converging to the induction of apoptosis and differentiation in APL cells (Supplementary Fig. S6), thus providing new insights to the mechanisms of EGCG in leukaemia. Hence, as EGCG clinical effects has been reported in hematologic malignancies such as chronic lymphocytic leukaemia^7–9^, EGCG, associated or not with chemotherapies, could represent a promising option for patients ineligible for current mainstay treatments.

## Methods

### APL model

Mice were bred and maintained under pathogen-free conditions at the University of Campinas. Cells from leukaemia hCG-PML/RAR transgenic mice were resuspended in RPMI supplemented with 3% FBS. After 4–6 h 2Gy irradiation, 1×10^6^ cells were injected in the caudal vein of 12‒16-week-old female NOD.CB17-Prkdc^scid^/J mice, 18–20 g (The Jackson Laboratory, USA). Twelve days after transplantation, animals were diagnosed with leukaemia, characterized by leucocytosis (leukocytes >30 × 10,000/µL) and/or anaemia (haemoglobin levels < 10 g/dL) and/or thrombocytopenia (platelets < 500 × 10,000/µL) plus the presence of at least 1% of peripheral blood blast^23–25^. Mice were then randomly selected (n = 10 per group) to receive i.p. administration of EGCG (25mg/kg/day) (Cayman Chemical Co.) or vehicle (saline) for five consecutive days. At the end of the treatment (16th day), peripheral blood was collected for analysis and mice were then deepening anaesthetized for the sacrifice. Bone marrow cells were obtained using PBS flushing, and splenic cells were obtained by mechanical disruption with PBS. For survival analysis, mice (n = 10 per group) were submitted to the same treatment until the date of death. All experiments were conducted according to National Institutes of Health guide for the care and use of Laboratory Animals and ARRIVE guidelines, and were approved by the Ethics Committee for Animals of the University of Campinas (number 3995-1/A).

### Flow cytometry analysis

Cell suspensions from bone marrow, peripheral blood or spleen were stained with specific antibodies for 20 min at room temperature in order to characterize cell populations. The antibodies employed were anti-CD45 PERCP (pan leukocyte marker), anti-CD34 APC (hematopoietic stem cells), anti-CD117 FITC (immature myeloid cells), anti-CD3 APC (lymphocyte), anti-CD11b PE (mature myeloid cells, monocyte/macrophage), and anti-Gr-1 FITC (mature myeloid cells, neutrophil). A total of 30,000 events/sample were acquired on a FACSCalibur cytometer and analysed using FlowJo software.

### Apoptosis assays

Total bone marrow and spleen cells were resuspended in Annexin-V binding buffer (BD Pharmingen, San Diego, CA, USA) containing 1 µg/mL APC labelled Annexin-V and incubated for 15 min at room temperature in the dark. Propidium iodide was added 10 min before flow cytometry analysis. Samples were acquired on a FACSCalibur cytometer and analysed using FlowJo software.

### Measurement of intracellular ROS

Bone marrow cell suspensions were first stained with the antibodies anti-CD34 APC, anti-CD45 PERCP, anti-CD117 APC and anti-Gr-1 PE; and then incubated with 25 µmol/L of 2’,7’-dichlorofluorescein diacetate (DCFDA)-FITC for 30 min at 37 °C. Samples were acquired on a FACSCalibur cytometer and analysed using FlowJo software.

### Western blot analysis

Protein was extracted in RIPA buffer from total bone marrow or spleen cells and quantified using Bradford reagent. Equal protein amounts were loaded on 8–15% SDS polyacrylamide gels and electrophoretically transferred to a nitrocellulose membrane. Nonspecific binding sites were blocked by incubation with buffer containing Tris (10 mmol/L, ph7.4), NaCl (150 mmol/L), Tween 20 (0.1%), and fat-free powdered milk (5%). Membranes were incubated overnight with a specific antibody at 4 ºC, followed by horseradish peroxidase conjugated secondary antibody, for one hour at room temperature. Immunoreactivities were visualized by ECL Western Blot Analysis System (Amersham Pharmacia Biotech). Quantification of band intensity was performed by UN-SCAN-IT gel 6.1 (Silk Scientific, Orem, UT, USA). The antibodies employed were ACTIN (sc-1616), AKT1/2/3 (sc-8312), BAD (sc-8044), BAX (sc-20067), BCL-2 (sc-492), PML (sc-5621), c-JUN (sc376488), CYCLIN D1(sc8396) from Santa Cruz Biotechnology (Texas, CA, USA); PIN1 (#3722), NFκB p65 (#4764) from Cell Signaling Technology (Beverly, MA, USA); PML+RARα (ab43152), c-MYC (ab32072), laminin receptor (67LR or 67kDa; ab137388) from Abcam (Cambridge, MA, USA).

### Imaging flow cytometry

Bone marrow cell suspensions were stained with the antibodies anti-CD117 APC, anti-CD34 APC, PML Alexa-488 and 7-AAD (nuclear stain), from BD biosciences (San Jose, CA, USA) and fixed with BD Cytofix/Cytoperm Kit. A total of 5,000 events were acquired on the ImageStreamX (Amnis/EMD Millipore, Seattle, WA, USA) and the image analysis was performed using IDEASVR software^31^.

### Cell culture and differentiation assay

Human APL cell line, the NB4, obtained from the Deutsche Sammlung von Mikroorganismen and Zellkulturen Gmbh (DSMZ, Braunschweig, Germany) were cultured in RPMI supplemented with 10% fetal bovine serum (FBS), penicillin/streptomycin and amphotericin B, and maintained at 37 °C in a 95% humidified atmosphere (incubator), containing 5% CO_2_. 1 × 10^5^/ml cells were seeded into petri dishes and they were treated with ATRA (1 µM), or EGCG (12.5 and 20 µg/ml) in the presence or absence of NAC (10 mM), from Sigma Chemical Co. (St. Loius, MO, USA) for three hours. After 96h, the expression of the differentiation markers was determined by flow cytometric analysis; the antibodies employed were anti-CD11b FITC, CD14 PE, CD15 APC and CD66 PE. For apoptosis assays, the NB4 cells was seeded as described above and treated with EGCG (12.5–50 µg/ml) for 48h in the presence or absence of NAC (10 mM).

### Statistical analysis

Statistical analyses were performed using GraphPad Instat 5 (GraphPad Software, Inc., San Diego, CA, USA). For comparisons, the Student *t* test was used. Results are presented as mean ± SD. A P value of < 0.05 was considered as statistically significant. Survival of the PML/RARα mice treated with EGCG was analysed by Log-Rank (Mantel-cox) test.

## Supplementary Information

Supplementary Information
