# Supplementary material for: (–)-Epigallocatechin-3-gallate induces apoptosis and differentiation in leukaemia by targeting reactive oxygen species and PIN1
Source: Sci Rep. 2021 Apr 27;11:9103. doi: 10.1038/s41598-021-88478-z (PMC8079435; doi:10.1038/s41598-021-88478-z)
Supplement: Supplementary file 1 — Supplementary Information [file 41598_2021_88478_MOESM1_ESM.pdf]

(-)-Epigallocatechin-3-gallate induces apoptosis and differentiation in leukaemia by targeting reactive oxygen species and Pin1

Fernanda Isabel Della Via[1], Rodrigo Naoto Shiraishi[1], Irene Santos[1], Karla Priscila Ferro[1], Myriam Janeth Salazar Terreros[1], Gilberto Carlos Franchi Junior[2], Eduardo Magalhães Rego[3], Sara Teresinha Olalla Saad[1], and Cristiane Okuda Torello[1,*]

[*]corresponding author: cris.okuda@gmail.com

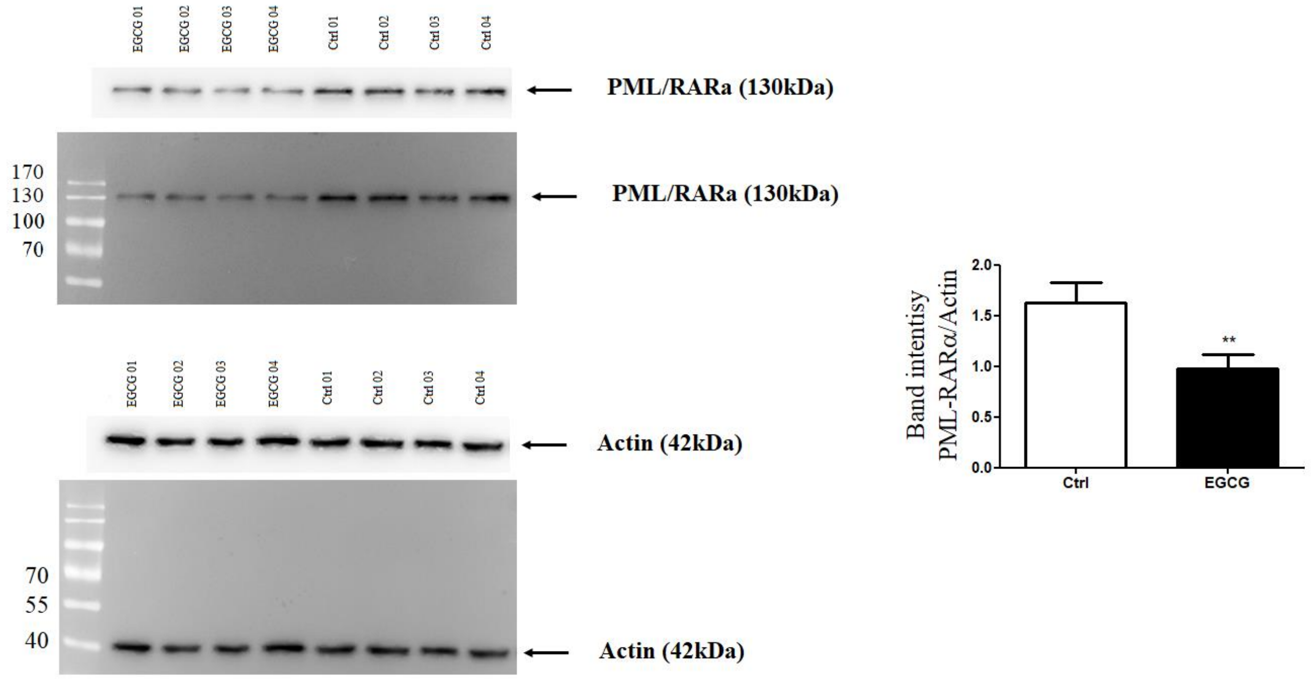

SUPPLEMENTARY FIGURE S1

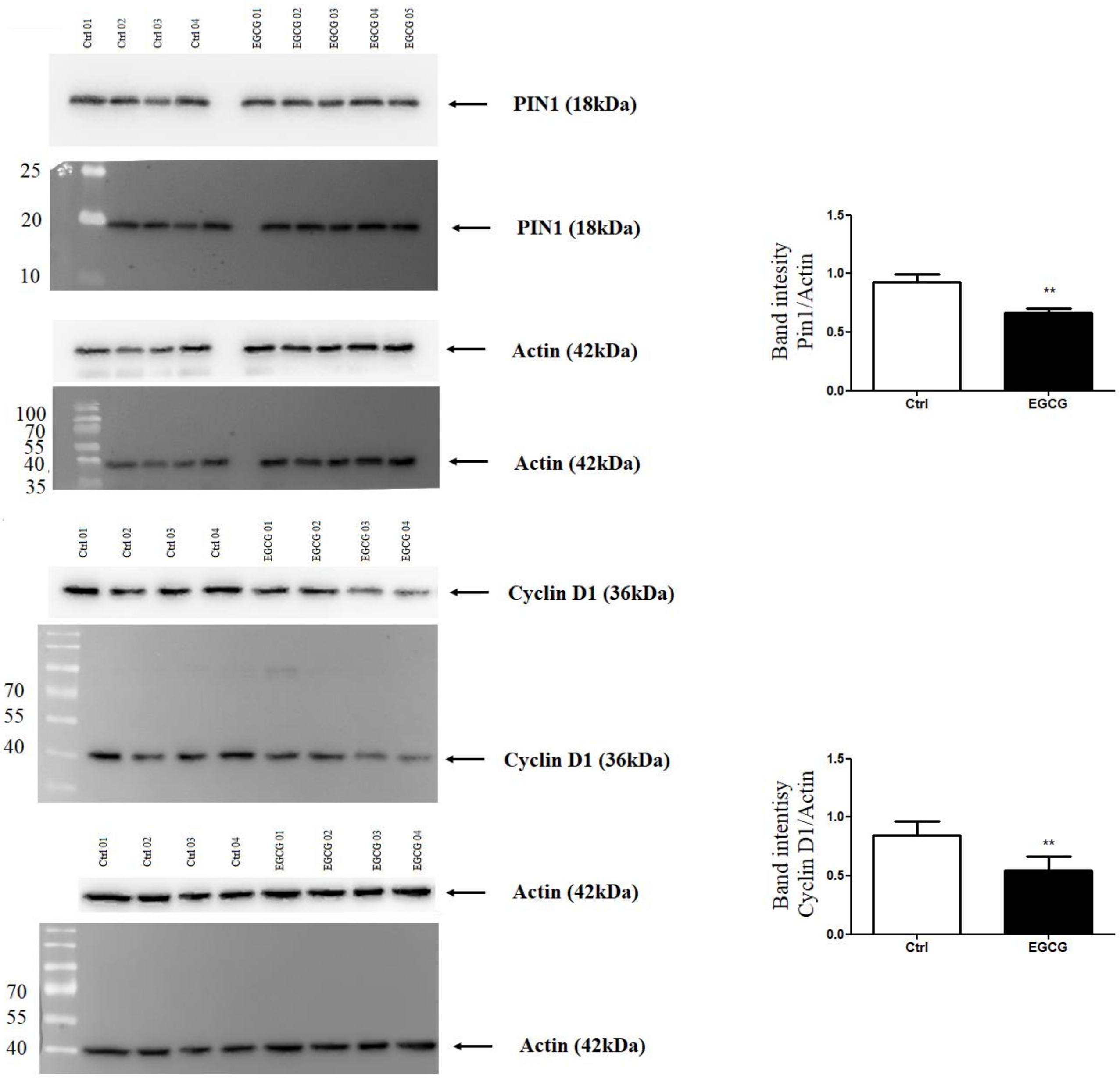

SUPPLEMENTARY FIGURE S2

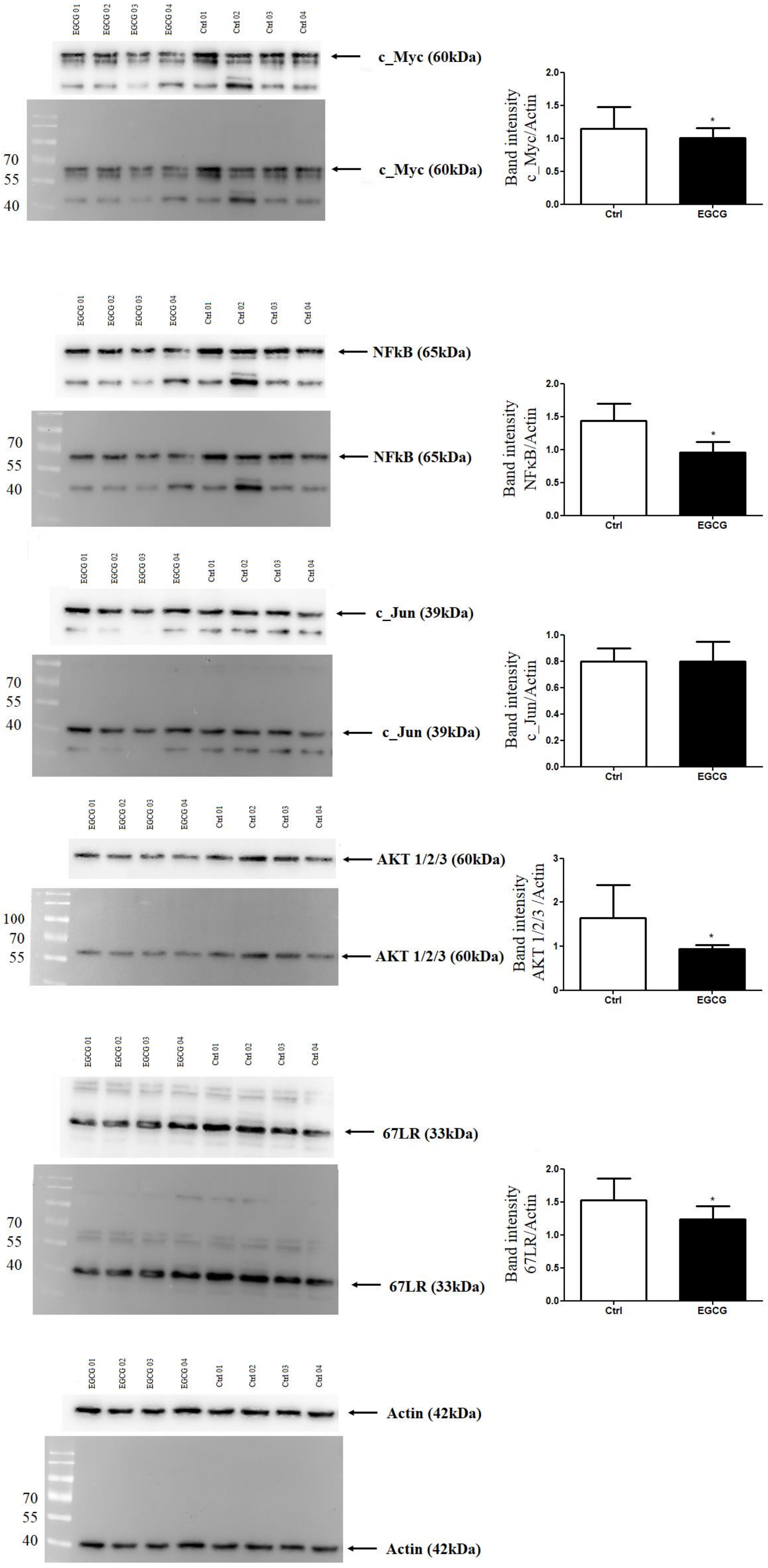

SUPPLEMENTARY FIGURE S3

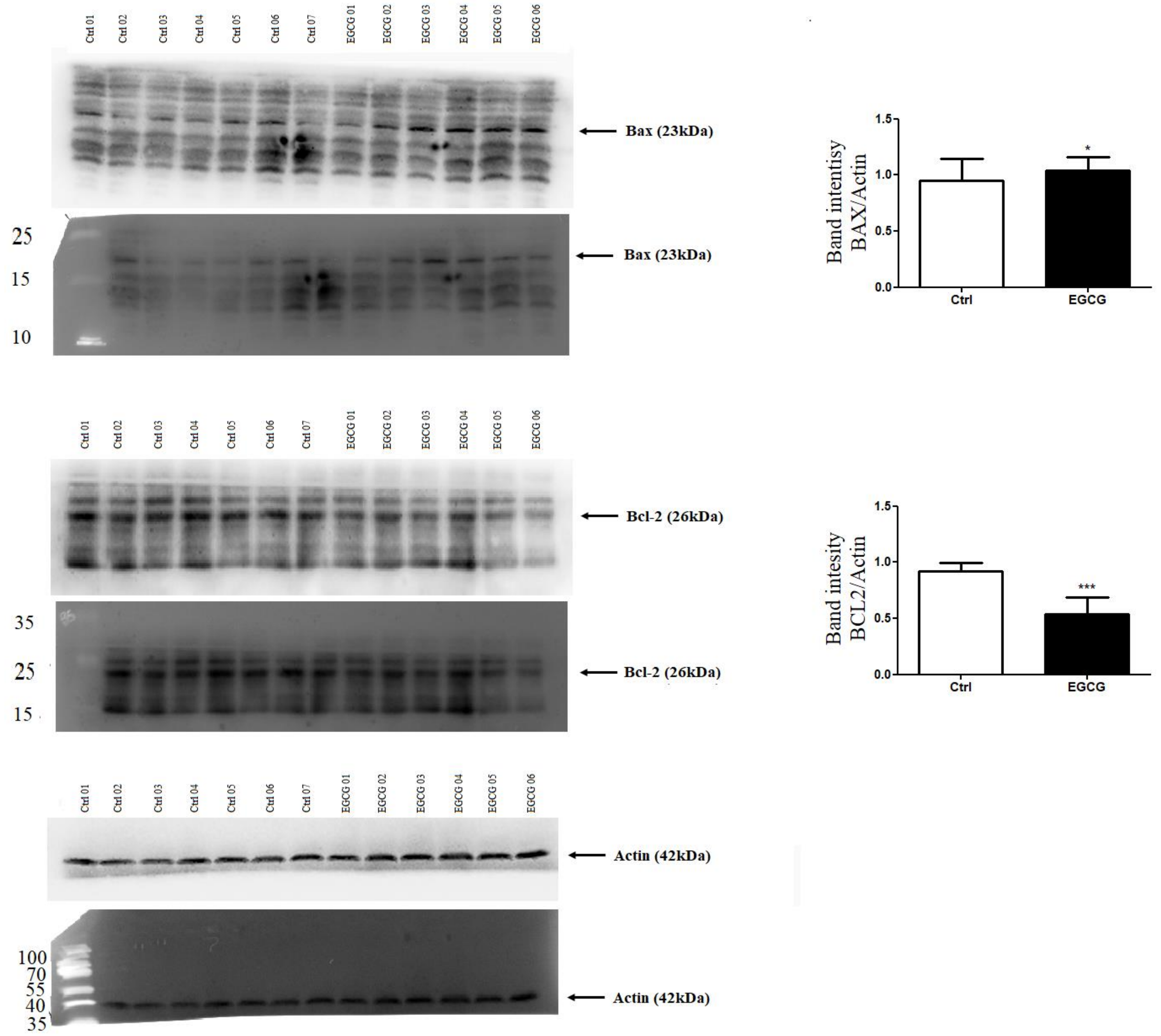

SUPPLEMENTARY FIGURE S4

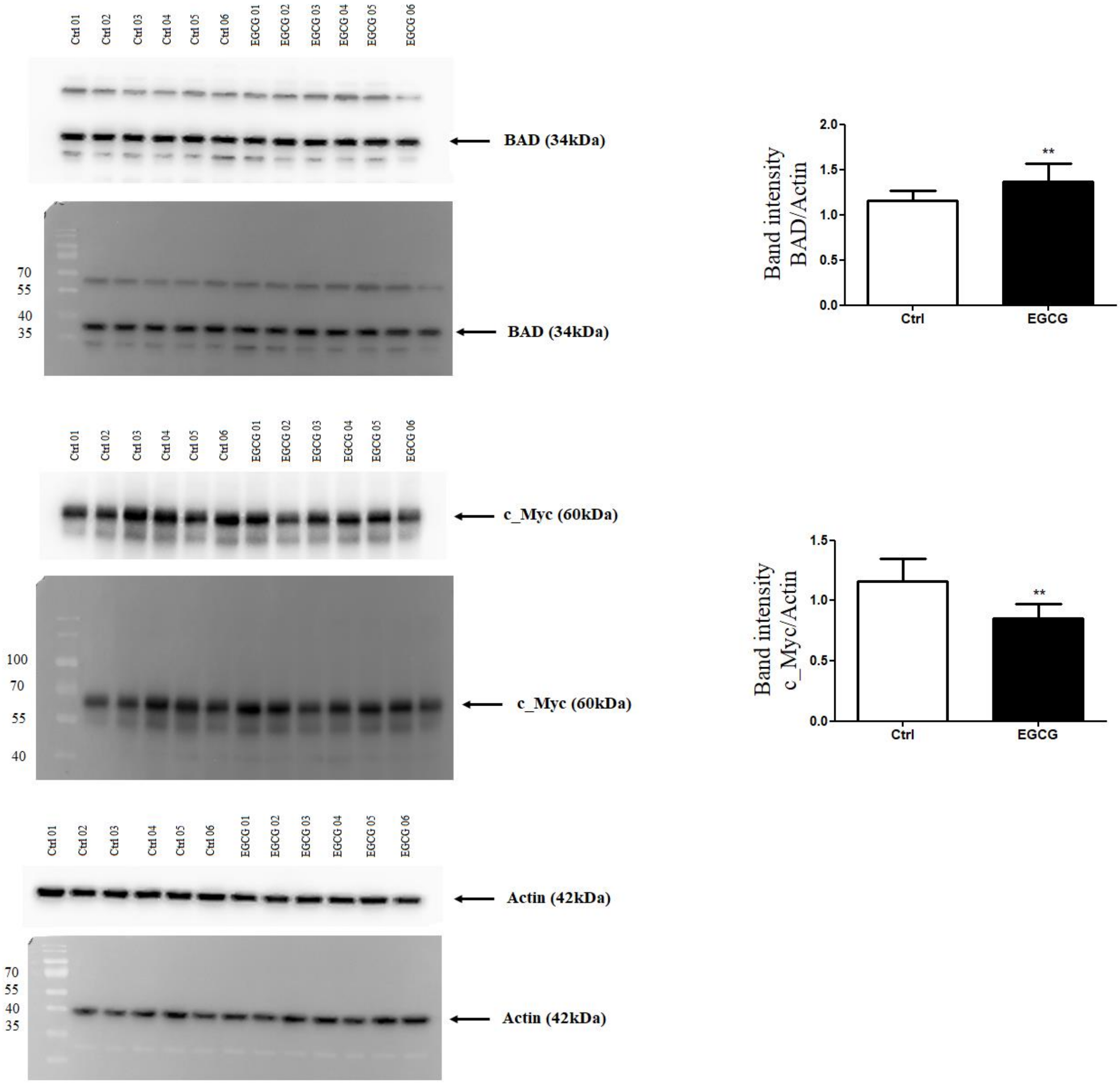

SUPPLEMENTARY FIGURE S5

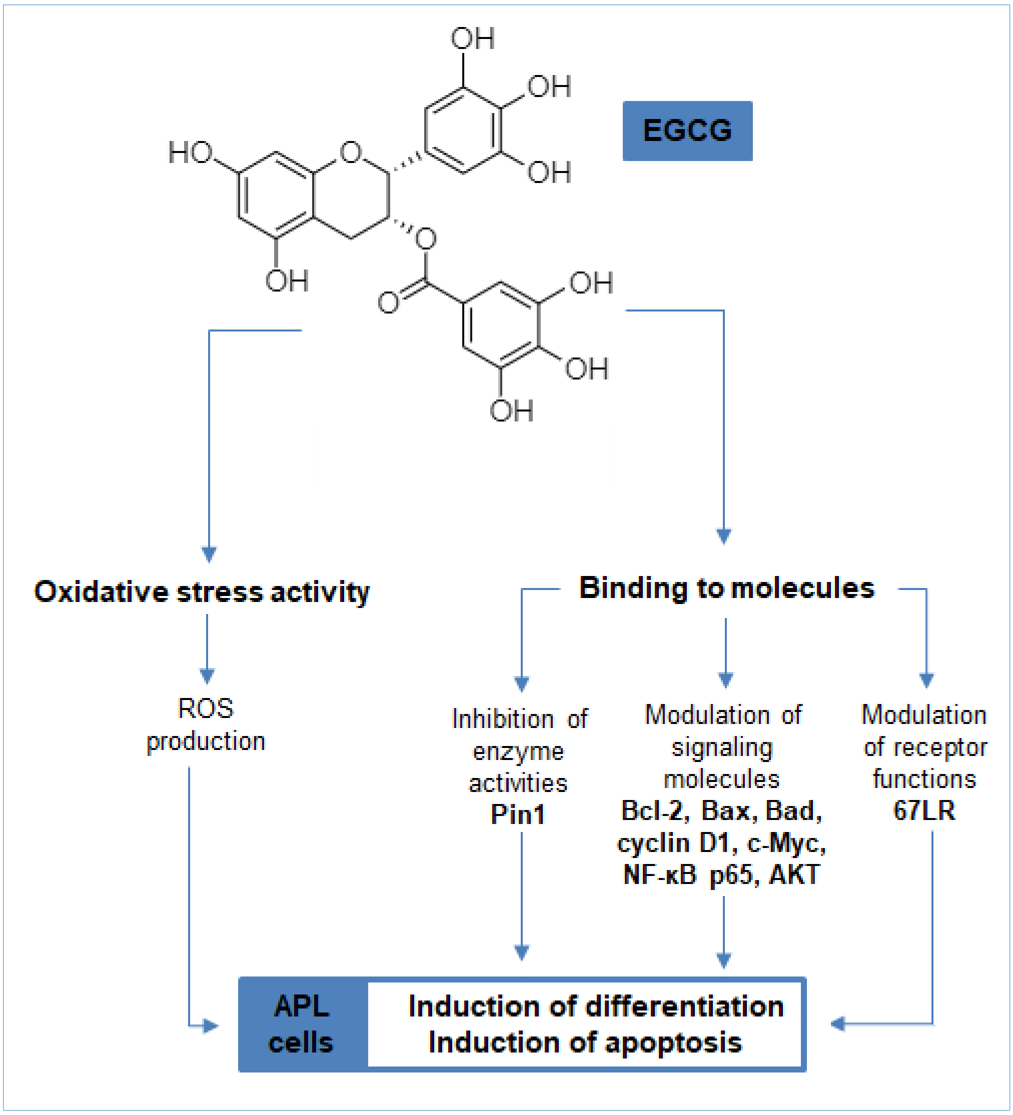

SUPPLEMENTARY FIGURE S6
